# Macro- and microcirculation characteristics in the territory of the anterior cerebral artery in infants with congenital heart diseases

**DOI:** 10.1007/s00380-025-02549-z

**Published:** 2025-05-24

**Authors:** Yordan Hristov Georgiev, Mirjam Schöne-Leupolz, Johannes Nordmeyer, Christian Schlensak, Rafal Berger, Frank Fideler, Martin Ulrich Schuhmann, Julian Zipfel, Jörg Michel, Felix Neunhoeffer

**Affiliations:** 1 Department of Pediatric Cardiology, Pulmonology and Pediatric Intensive Care Medicine, University Children’s Hospital Tübingen, Hoppe-Seyler-Str. 1, 72076 Tübingen, Germany; 2 Department of Thoracic and Cardiovascular Surgery, University Hospital Tübingen, Hoppe-Seyler-Str. 1, 72076 Tübingen, Germany; 3 Department of Anaesthesiology and Intensive Care Medicine, University Hospital Tübingen, Hoppe-Seyler-Str. 1, 72076 Tübingen, Germany; 4 Section of Pediatric Neurosurgery, Department of Neurosurgery, University Hospital Tübingen, Hoppe-Seyler-Str. 1, 72076 Tübingen, Germany

**Keywords:** Cerebral autoregulation, Cerebral macrocirculation, Cerebral microcirculation, Congenital heart disease, Cardiac surgery

## Abstract

Although cerebral macrocirculation is routinely assessed postoperatively in infants in the pediatric intensive care unit, monitoring cerebral microcirculation is not yet a standard practice. Our objective was to investigate the correlation between parameters of cerebral macro- and microcirculation in children following cardiac surgery and compare them with patients after neurosurgical and abdominal procedures. We conducted a prospective observational study in infants who underwent congenital cardiac surgery, visceral surgery, and neurosurgical procedures to measure parameters of cerebral macro- and microcirculation. Doppler ultrasound of anterior cerebral artery was performed, along with measurements of microcirculatory parameters using O2C device. 89 infants were included in the study. Group 1 (*n* = 35) comprised children after corrective cardiac surgery, group 2 (*n* = 22), after aortopulmonary shunt procedures, group 3 (*n* = 11), after Glenn operations, and group 4 (*n* = 21), after abdominal or neurosurgical procedures. The systolic peak flow was significantly lower in groups 2 and 3 compared to groups 1 and 4, 52.3 and 56.7 versus 59.6 and 68.8 cm/s, *p* = 0.01, respectively. Pulsatility index was higher in patients of group 2 compared to groups 1, 3 and 4, 2.5 vs. 1.3, 1.4, and 1.5 (*p* < 0.001), respectively. The cerebral blood flow in the staged palliation groups (2 and 3) was lower compared to groups 1 and 4, 203 and 236 vs. 250 and 262 AU, *p* = 0.045. Children undergoing staged palliation may show variations in cerebral macro- and microcirculation. Both approaches described in our study provide complementary information and can accordingly be utilized in the postoperative intensive care period. Future studies should focus on establishing reference values for macro- and microcirculation parameters across various patient populations.

## Introduction

As one of the most prevalent congenital malformations in human population, the incidence of congenital heart diseases (CHD) reaches 1.2% [1, 2]. More than 13 million people with CHD were living in 2019 worldwide [3]. Successful treatment can be offered nowadays to the majority of patients with CHD, allowing over 90% of these patients to reach adulthood [4]. Increasing numbers of children with CHD are reaching nowadays adulthood. Measures of operative success are no longer limited to survival but also to quality of life and long-term neurologic outcomes. Consequently, the neurological outcomes of these patients are becoming increasingly important as many neonates with complex CHD are at risk to suffer severe brain damage due to hypoxemia occurring prior to therapeutic measures or in the perioperative period [5, 6]. Several studies demonstrated that children who underwent congenital cardiac surgery may suffer from up to 55% neurological complications [7–9].

Since established methods exist for monitoring cerebral macrocirculation, such as transcranial Doppler ultrasound of the middle cerebral artery, microcirculation is yet not routinely monitored [10]. Several methods were employed in recent years in an effort to monitor cerebral blood flow (CBF) and thus microcirculation.

Near-infrared spectroscopy (NIRS) is a widely used, non-invasive, and continuous method for real-time determination of autoregulation integrity, increasingly utilized in the clinical practice over the past two decades [11–13]. Although some surrogate parameters for non-invasive evaluation of CBF such as cerebral regional oxygen saturation have been recognized, the evidence of the impact of this technology remains uncertain [14].

An alternative and easy-to-use device called O2 C (oxygen-to-see device, LEA Medizintechnik, Giessen, Germany) can provide real-time information about cerebral microcirculation. The O2 C device is based on a combination of white light spectrometry and laser-Doppler flowmetry, allowing continuous determination of post-capillary regional oxygen saturation (cSO_2_), capillary–venous cerebral blood flow (CBF), and relative hemoglobin content (cHb) in the investigated tissue [15]. The procedure was first described from Walter et al. and validated in the pig brain [16]. These surrogate parameters enable real-time assessment of brain microcirculation.

The objective of our study was to investigate the effects of congenital heart diseases on CBF and oxygen delivery postoperatively and to explore potential correlations between cerebral macro- and microcirculation using O2 C device.

## Materials and methods

### Study design and setting

Between July 2015 and June 2017, we conducted a prospective, observational, non-randomized study at the 14-bed tertiary PICU of the University Children's Hospital, Tübingen, Germany. Infants who underwent congenital heart surgery with cardiopulmonary bypass (CPB), as well as children who underwent visceral surgery and neurosurgical procedures were included. The study had obtained official approval from the Ethics Committee at the Faculty of Medicine, University of Tübingen (574/2012BO1). Prior to inclusion in the study, approval from both parents was obtained.

### Study protocol and definitions

O2 C and color duplex sonography were performed by the same operator 12–24 h after the admission at the PICU. All patients received treatment according to a standardized protocol. Patients were included if they had a stable cardiac and respiratory function, without any significant changes in heart rate and arterial blood pressure for at least 30 min under current inotropic and vasoactive medication. Additionally, they should not have required fluid therapy, experienced bleeding demanding transfusion at the PICU, or had abnormal arterial CO_2_ levels. The oxygen saturation had to be in accordance with the predefined targets [17]. Postoperative hemodynamic therapy was managed with norepinephrine, milrinone, and/or adrenaline. The hemodynamic status was actively monitored using invasive arterial blood pressure, central venous saturation, serum lactate, and diuresis parameters, while perfusion status was assessed through capillary refill time monitoring. Therapeutic goals were MAP > 45 mmHg in children < 6 months and MAP > 50 mmHg in children > 6 months, a difference between arterial oxygen saturation and central venous saturation of 30%, diuresis > 2 mL/kg/h, capillary refill time < 2 s, and lactate < 2 mmol/L.

To assess cerebral microcirculation, measurements were conducted using the device"Oxygen to See"(O2 C, LEA Medizintechnik GmbH, Giessen). Before the measurement consistent initial conditions were established, including calibration, white balance, and ambient light correction. A probe with a penetration depth of approximately 15 mm was utilized. Cerebral hemoglobin concentration (cHb), cerebral oxygen saturation (cSO_2_), cerebral microvascular blood flow (CBF), and cerebral microvascular blood flow velocity (CBV) on both the right and left forehead were measured. To avoid motion and pressure artifacts, only children in a calm state were investigated, and the probes were manually fixed to the head with minimal movement and pressure. The study parameters were recorded for 30 s at each site. A standardized, nurse-driven, goal-oriented protocol for pediatric analgesia and sedation has been implemented in our PICU [18]. To assess levels of analgesia and sedation, the validated COMFORT-B scale (target range: 12–18 points) and the Nurse Interpretation of Sedation Score (target score: 2) are routinely used [19–21]. As the dark and hairy skin can interfere with the signal, measurements were performed on hairless areas of the forehead. If necessary, the skin was cleaned of colored disinfectants. The optimal operating temperature of 15–30 °C was maintained for all measurements.

All patients underwent a B-mode transfontanelle cerebral ultrasound using a micro-convex transducer C10-3 (Mindray®) of computed sonography system (ZONARE© Medical Systems Inc., 2014, Mountain View, USA) to exclude a cerebral hemorrhage. After that, an angle-corrected color duplex sonography of the anterior cerebral artery (ACA) in the midsagittal plane was performed by the same operator. Because the two anterior cerebral arteries run very close together, they generally cannot be differentiated and are therefore considered as a single vessel. In front of the third ventricle, the ACA runs directly toward the transducer, which results in an optimal insonation angle for reliable flow measurement. In this region, six parameters were measured using color duplex sonography: systolic peak flow, end-diastolic flow velocity, minimal and maximal time average velocity (TA_max_ and TA_min_), as well as the resistance index (RI) and the pulsatility index (PI).

RI was calculated using the following formula: RI = (TA_max_ − TA_min_)/TA_max_. PI was calculated using the following formula: PI = (TA_max_ − V_min_)/V_mean_.

The vasoactive–inotropic score (VIS) was calculated using the following formula: 100 × epinephrine dose [μg/kg/min] + 50 × Levosimendan dose [μg/kg/min] + 10 × Milrinone dose [μg/kg/min] + 100 × Norepinephrine dose [μg/kg/min] [22].

The children were grouped into six categories according to the RACHS-1 score (Risk Adjustment for Congenital Heart Surgery). It was established in 2002 as a method for assessing the postoperative mortality risk based on the surgical procedure in children with congenital heart defects [23]. In neither of the surgeries was deep hypothermic circulatory arrest performed.

### Statistical analysis

Statistical analysis was performed using SigmaPlot (Version 13 for Windows, Systat Software, Inc., San Jose, CA, United States). Continuous data are presented as median and interquartile range (IQR) and categorical data are presented as frequencies and percentages. All *p* values < 0.05 were considered statistically significant. The variables are represented using box plots, showing the data between the first and third quartiles. A regression analysis was conducted to investigate the relationship between macro- and microcirculation parameter.

## Results

89 infants, who had recently undergone surgery, were included in the study. Of these, 68 patients underwent congenital cardiac surgery with CPB, and 21 patients underwent abdominal or neurosurgical procedures (group 4). The cardiac operations were further divided into three subgroups: group 1 with 35 children after corrective cardiac surgery, group 2 with 22 children after aortopulmonary shunt procedures and group 3 with 11 children after Glenn operations. Demographic and clinical characteristics of the children are represented in Table 1.

**Table 1:** Patient characteristics and clinical data

	Group 1 (*N* = 35)	Group 2 (*N* = 22)	Group 3 (*N* = 11)	Group 4 (*N* = 21)	*p* value
Gender m (*n*)	19	13	7	11	0.92
Weight (kg)	5.1 (3.5–8.5)	3.5 (2.8–4.3)	6.8 (5.2–8.5)	5.1 (2–9.9)	< 0.001*
Age (days)	128 (9–318)	13 (6–51)	246 (118–355)	103 (1–316)	< 0.001*
VIS	7.4 (0–23.8)	8.8 (0–15.4)	4.7 (0–10.2)	0 (0–8.3)	< 0.001*
CPB (min)	104 (0–229)	180 (43–242)	45 (35–83)	–	< 0.001*

Results are represented as median (IQR). * represents statistically significant results

The diagnoses of the patients in group 1 included atrial septal defect (*n* = 2), atrioventricular septal defect (*n* = 11), ventricular septal defect (*n* = 9), aortic arch anomalies (*n* = 4), Tetralogy of Fallot (*n* = 4), pulmonary atresia (*n* = 2), total anomalous pulmonary venous connection (*n* = 1), anomalous left coronary artery from the pulmonary artery (*n* = 1), and truncus arteriosus type 2 (*n* = 1). The diagnoses of the patients in group 2 included hypoplastic left heart syndrome (*n* = 11), tricuspid atresia (*n* = 4), and pulmonary stenosis or atresia (*n* = 7). The diagnoses of the patients in Group 3 included right-dominant unbalanced atrioventricular septal defect (*n* = 2), univentricular heart (*n* = 3), and hypoplastic left heart syndrome (*n* = 6).

**Fig. 1:**
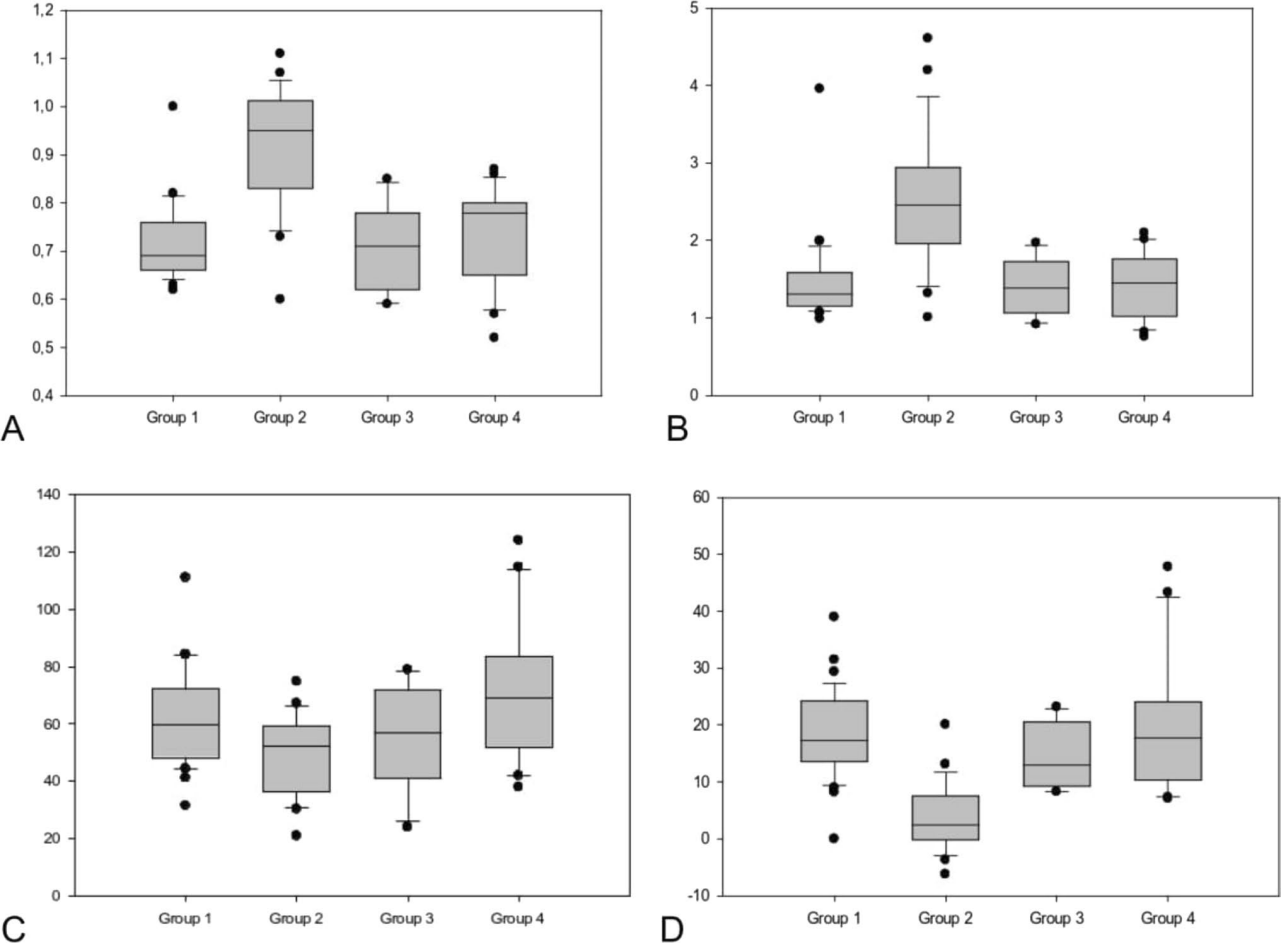
Box plot analysis of the parameters of macrocirculation in all four subgroups represented as median and IQR. **A** RI in ACA—a. cerebri anterior, **B** PI in ACA, **C** PSV in ACA, **D** ED in ACA. RI, resistance index, P, pulsatility index, PSV, systolic peak flow, ED, end-diastolic flow velocity

Table 2 represents the subgroup analysis of parameters related to macro- and microcirculation. Due to the different hemodynamic nature after staged palliation in groups 2 and 3, the PSV was significantly lower compared to groups 1 and 4—52.3 and 56.7 versus 59.6 and 68.8 cm/s, *p* = 0.01, respectively (Fig. 1C). Since surgical procedures in patients with functionally univentricular hearts always included placement of an aortopulmonary shunt resulting in diastolic run-off from the systemic circulation, EDV was significantly lower in patients of Group 2 compared with patients of Group 1 and 4—2.4 vs. 17.4 and 17.7, *p* < 0.001 (Fig. 1D). Accordingly, PI was higher in patients of Group 2 compared to Groups 1, 3 and 4, 2.5 vs. 1.3, 1.4 and 1.5, respectively (Fig. 1B).

**Table 2:** Parameter of cerebral macro- and microcirculation in Groups 1–4

	Group 1 (*N* = 35)	Group 2 (*N* = 22)	Group 3 (*N* = 11)	Group 4 (*N* = 21)	*p* value
Cerebral macrocirculation					
RI	0.7 (0.6–1)	1 (0.6–1.1)	0.7 (0.6–0.9)	0.8 (0.5–0.9)	< 0.001*
PI	1.3 (1–4)	2.5 (1–4.6)	1.4 (0.9–2)	1.5 (0.8–2.1)	< 0.001*
PSV [cm/s]	59.6 (31.4–111)	52.3 (20.9–74.8)	56.7 (23.9–78.9)	68.8 (37.9–124)	0.01*
EDV [cm/s]	17.4 (0–39)	2.4 (− 6.2 to 20.1)	12.9 (8.3–23.2)	17.7 (7.1–47.8)	< 0.001*
TA_max_ [cm/s]	29.5 (12.4–64.3)	18.2 (8.3–41.3)	24.8 (15.6–43.4)	37.7 (16.9–78)	< 0.001*
TA_min_ [cm/s]	13.5 (5.3–32.7)	8.2 (4–20.6)	12 (5.7–18)	15 (8–35.1)	< 0.001*
Cerebral microcirculation					
CBF [AU]	250 (96–520)	236 (126–360)	203 (141–318)	262 (179–322)	0.045*
cSO_2_ [%]	60 (45–84)	65 (50–83)	63 (22–78)	66 (40–85)	0.003*
CBV [AU]	69 (20–88)	65 (50–83)	63 (53–80)	74 (65–90)	0.001*
cHb [AU]	68 (33–106)	89,5 (57–130)	61 (48–94)	79 (42–140)	< 0.001*

Results are represented as median (IQR). * represents statistically significant results

**Fig. 2:**
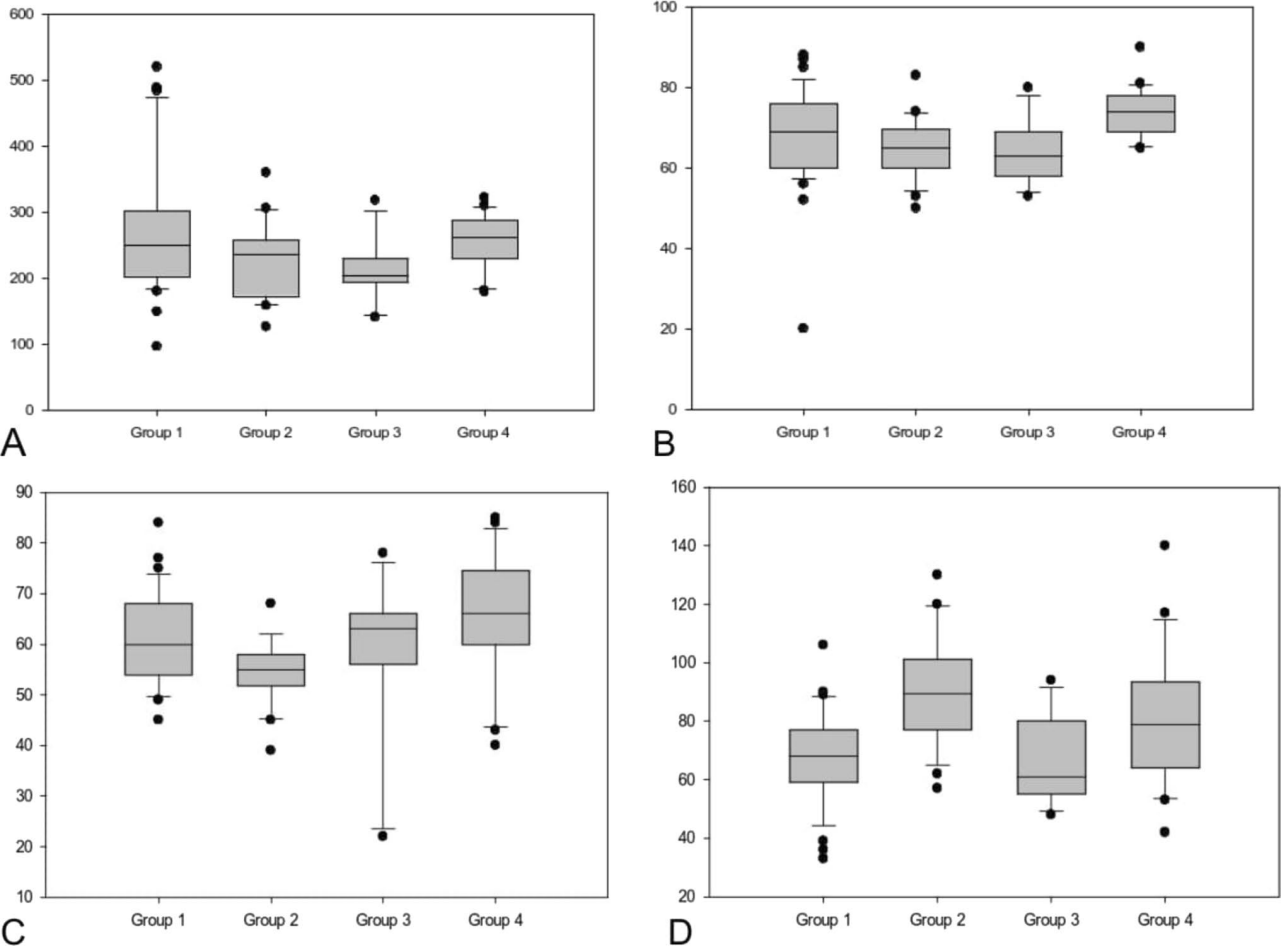
Box plot analysis of the parameters of microcirculation in all four subgroups represented as median and IQR. **A** CBF—cerebral microvascular blood flow, **B** CBV—cerebral microvascular blood flow velocity, **C** cSO2—cerebral oxygen saturation, **D** cHb—cerebral hemoglobin concentration

The parameters of microcirculation also exhibited differences across all subgroups. The lowest values of CBF and CBV were observed in Group 3 (Glenn group), followed by Group 2 (Shunt group) in comparison to Group 1 (corrective cardiac surgery group) and Group 4 (control group): CBF 203 and 236 vs. 250 and 262 AU, *p* = 0.045, CBV 63 and 65 vs. 69 and 74 AU, *p* = 0.001 (see Fig. 2).

**Fig. 3:**
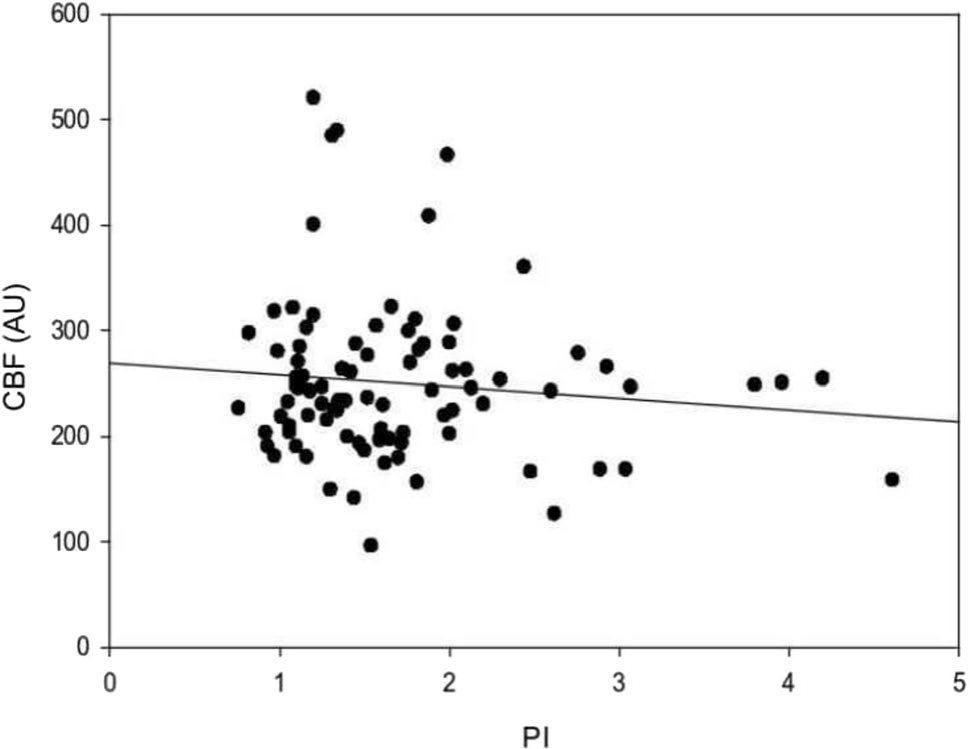
Linear relationship between PI of ACA and CBF for the entire population (*p* = 0.304, *R* = 0.110, *R*^2^ = 0.0121, Adj *R*^2^ = 0.0008)

The regression analysis of the entire population revealed no direct relationship between CBF and PI (*p* = 0.304; *R* = 0.110; *R*^2^ = 0.0121). (Fig. 3).

## Discussion

Over the last 20 years, there has been a growing interest on monitoring cerebral microcirculation. Efforts have been focused on identifying global microcirculatory surrogate parameters to measure changes in the cerebral blood flow and cerebral blood volume that are applicable in the clinical praxis [24, 25]. Microcirculation can be easily and non-invasively monitored at bedside by O2 C device [26, 27].

In our study, we aimed to investigate the utility of O2 C device to assess microcirculatory parameters in patients undergoing various surgeries. Despite its small patient size, which might have reduced the study's power, it provides valuable insights into the parameters of micro- and macrocirculation in children with different pathological conditions.

The autoregulation of CBF requires tight control, as excessive flow can lead to increased intracranial pressure, while insufficient flow can result in inadequate brain perfusion and subsequent cerebral ischemia [27]. The control occurs at multiple levels. Vascular reactivity and, consequently, microcirculation are influenced by several mechanisms, including the response to changes in perfusion pressure, vasoactive stimuli, local neuronal activity, and endothelium-dependent signaling [28]. If cerebral autoregulation is intact, fluctuations in macrocirculation do not directly affect microcirculation within certain limits. As the CBF passively fluctuates with arterial blood pressure when cerebral autoregulation is impaired, closely monitoring of the microcirculation might be crucial to avoid neurological complications.

A recent study [29] demonstrated that the use of noradrenaline to elevate blood pressure can increase cerebral blood flow (CBF) in critically ill patients. According to our study, the children in groups 1 and 2 had the highest vasoactive–inotropic scores (VIS) but the lowest CBF. Noradrenaline is the primary medication used to regulate postoperative blood pressure in children undergoing cardiac surgery, explaining the higher VIS values in these groups. However, our findings suggest that additional factors may play a crucial role in this complex process. One possible explanation is that altered hemodynamics in these patients may have a predominant influence. Another reason for impaired microcirculation in the patients of group 2 might be the higher RACHS-1 score, indicating a more complex surgical procedure. This was also demonstrated in another study by Suemori et al. [30]. They found that the decrease in cerebral tissue oxygenation after surgery was greater in higher-category RACHS-1.

A recent study demonstrated that cardiac output (CO) may also have an impact on cerebral blood flow [31]. It is well-established that the brain receives approximately 12% of the total cardiac output, highlighting its significant dependence on systemic circulation [32]. Despite the need to monitor CO and CBF in patients with reduced cardiac function, non-invasive methods to achieve this are not routinely integrated in clinical practice [33]. Consequently, maintaining hemodynamic stability and a near-physiological state appears to be the best approach to ensure adequate CBF. Moreover, it is advisable to set peri-operatively tight hemodynamic goals regarding arterial blood pressure to minimize excessive fluctuations in CBF, particularly when cerebral autoregulation is assumed to be impaired.

The overall reduced diastolic blood flow in patients who underwent staged palliation appears to contribute minimally to CBF. Two studies on preterm newborns demonstrated that CBF is primarily dependent on systolic, rather than diastolic, velocities [34, 35]. Therefore, this Doppler parameter may not provide a reliable assessment of impaired CBF, as also shown in our study.

In clinical practice, cerebral perfusion is typically assessed in large vessels, such as the middle cerebral artery, using transcranial Doppler sonography through temporal window [36]. In children, the open fontanelle provides a good acoustic window for performing an ultrasound examination. A major advantage of this method is that only the velocities in the vessels of interest can be examined. However, it is uncertain whether the altered flow profile in the large brain arteries, such as in patients with an aortopulmonary shunt, directly impacts microcirculation.

PI represents the compliance and resistance of the examined vessels [37]. PI is a complex function of various hemodynamic factors, including arterial pulse amplitude, cerebrovascular resistance, cerebral arterial compliance, and heart rate [38]. It is the most commonly used transcranial Doppler parameter in clinical practice and reflects cerebral perfusion pressure [38]. Thus, it might be used as a surrogate marker of the macrocirculation.

As demonstrated in the regression analysis in our study, the widely used transfontanelle cerebral duplex ultrasound may not entirely reflect the changes in the microcirculation. The PI did not show a linear correlation to CBF. Consequently, normal Doppler ultrasound findings do not definitely indicate intact microcirculation. It can only detect changes in large intracranial vessels, and not in the distal resistance vasculature. Probably, changes in the macrocirculatory Doppler parameters will only become noticeable at later stages of pathological processes. Thus, it may be beneficial to monitor parameters of cerebral microcirculation during hemodynamically unstable phases, such as the early postoperative period after congenital cardiac surgery. Neurological assessment of intubated patients through clinical examination is challenging. Therefore, non-invasive monitoring might be useful, especially for children who have undergone complex cardiac surgery, such as shunt procedures, and are not expected to be extubated immediately postoperatively. As shown in our study, the O2 C device can be reliably and easily implemented in the early postoperative period or during phases of hemodynamic instability. However, the impact of reduced CBF on further neurological development is still not well-understood. Many questions remain unanswered, such as when changes in the microcirculation become detectable through macrocirculatory parameters, how various physiological factors influence both micro- and macrocirculation, and whether these changes have long-term effects. Furthermore, the precise role of cardio-cerebral coupling is still not fully understood. Therefore, further research in this field is urgently required, probably through interventional studies.

Our study has several limitations. It was a single-center study with relatively small sample size. Both the O2 C measurements and the Doppler ultrasound data were recorded over a brief period of 30 s, thus representing snapshots in time. The aim of the study was to provide initial insights into the use of the O2 C device in children with congenital heart disease. Additionally, we aimed to compare it with parameters of macrocirculation, which are also snapshot measurements. Consequently, it was decided to first examine the steady state. Future investigations should focus on including continuous measurements of microcirculation also in hemodynamically instable states.

Additionally, comorbidities were not considered, which could impact the results. Previous surgeries, congenital abnormalities, or unidentified genetic syndromes may influence cerebral perfusion changes. Furthermore, this study was conducted on patients who received different doses of sedatives, which may have additionally influenced microcirculation. The evidence on the impact of sedatives on cerebral blood flow remains controversial [39–41]. However, this aspect was not investigated in our cohort and represents another limitation of the study.

## Conclusion

Macro- and microcirculatory parameters in children undergoing staged palliation differ significantly from those observed in patients undergoing corrective cardiac surgery, vascular surgery, and neurosurgery. This may reflect the reduced compensatory reserves in this population and highlights the need of closer monitoring of microcirculation in the early postoperative period. Further analysis is needed to better understand the significance of these findings and their impact on patient outcomes. This can only be achieved through interventional multi-central studies aimed at determining thresholds, beyond which patients may benefit from intervention.

## Data Availability

The data that support the findings of this study are available from the corresponding author upon reasonable request. The data are not publicly available due to privacy or ethical restrictions.
